# Somatic symptom profile in patients with chronic heart failure with and without depressive comorbidity

**DOI:** 10.3389/fpsyt.2024.1356497

**Published:** 2024-03-19

**Authors:** Thomas Müller-Tasch, Bernd Löwe, Lutz Frankenstein, Norbert Frey, Markus Haass, Hans-Christoph Friederich

**Affiliations:** ^1^ Department of Psychosomatic Medicine and Psychotherapy, Klinikum am Weissenhof, Weinsberg, Germany; ^2^ Department of General Internal Medicine and Psychosomatics, University of Heidelberg, Heidelberg, Germany; ^3^ Department of Psychosomatic Medicine and Psychotherapy, University Medical Centre Hamburg-Eppendorf, Hamburg, Germany; ^4^ Department of Cardiology, University of Heidelberg, Heidelberg, Germany; ^5^ Department of Cardiology, Theresien Hospital Mannheim, Mannheim, Germany

**Keywords:** chronic heart failure, depression, somatic symptoms, Patient Health Questionnaire, treatment

## Abstract

**Background:**

Patients with chronic heart failure (CHF) frequently suffer from depressive comorbidity. CHF and depressive comorbidity can cause somatic symptoms. The correct attribution of somatic symptoms is important. Thus, we aimed to assess potential differences in somatic symptom severity between CHF patients with and without depressive comorbidity.

**Methods:**

We evaluated depressive comorbidity using the Patient Health Questionnaire-9 (PHQ-9), somatic symptom severity with the Patient Health Questionnaire-15 (PHQ-15), and sociodemographic and medical variables in 308 CHF outpatients. To compare somatic symptom severity between CHF patients with and without depressive comorbidity, we conducted item-level analyses of covariance.

**Results:**

Of the 308 participating patients, 93 (30.3%) met the PHQ-9 criteria for depressive comorbidity. These patients did not differ from those without depressive comorbidity with regard to age, sex, left ventricular function, and multimorbidity. Patients with depressive comorbidity scored significantly higher on ten out of thirteen PHQ-15 items than patients without depressive comorbidity. The largest effect sizes (0.71-0.80) were shown for symptoms of headache, chest pain, shortness of breath, and palpitations, and the latter three were potentially attributable to heart failure.

**Conclusions:**

Among patients with CHF, somatic symptoms are more pronounced in those with depressive comorbidity than those without depressive comorbidity. This finding is especially true for cardiac symptoms independent of CHF severity. The potential interpretation of somatic symptoms as correlates of depressive comorbidity must be recognized in clinical practice.

## Introduction

Chronic heart failure (CHF) is a common and long-lasting disease, and its incidence increases with age. The quality of life of CHF patients is severely limited, and their prognosis is poor (1). Depressive comorbidity is present in approximately 20% of CHF patients (2). This has a negative impact on the course and prognosis of CHF (1–3) via pathophysiological processes (4), and depression has negative effects on self-care behavior (5, 6). Therefore, the identification of depressive comorbidities in patients with CHF is very important and widely recommended in heart failure guidelines (1, 7). A majority of patients with depressive disorder present with somatic symptoms as their primary or even their only complaint, independent of a comorbid medical disease (8–11). Previous studies have shown that patients with CHF and comorbid depression experience a greater cardiac symptom burden than CHF patients without depressive comorbidity (12). The core symptoms of heart failure include ankle swelling, breathlessness, and fatigue (1). The latter two symptoms are unspecific and are also often mentioned by patients with depression (9, 10). In a previous study, we found no difference in the severity of somatic depression symptoms between depressed patients with or without CHF, which means that somatic depression symptoms are valid indicators of depression in CHF patients (5). In contrast, depressed patients with CHF reported lower levels of cognitive depression symptoms of “feeling down, depressed or hopeless” and “feeling bad about yourself” than depressed patients without CHF (5). This differentiation is important, as somatic-affective symptoms of depression but not cognitive-affective symptoms of depression are related to reduced quality of life (13) and increased mortality in patients with CHF (14). The explicit attribution of somatic symptoms to somatic or depressive origins can be difficult. Nevertheless, identifying a high somatic symptom burden is important, as it can be associated with functional impairment, decreased quality of life, and increased health care use (15). Additionally, somatic symptoms are predictors of the persistence of depressive disorders (16). As mentioned above, the studies described in the review by Katon et al. only investigated and reported more frequent and bothersome heart failure symptoms in depressed patients with CHF than in nondepressed patients with CHF (12). Other somatic symptoms, such as pain or abdominal symptoms, were not evaluated in these studies. In a previous study evaluating patients with coronary heart disease, the somatic symptom burden was high not only for cardiac symptoms but also for pain and difficulty in sleeping. A clear association between somatic symptom burden and depressive symptoms was also found (17). To our knowledge, no such study has been conducted in patients with CHF, which would be highly interesting for the aforementioned reasons.

Therefore, this cross-sectional study aimed to assess potential differences in the severity of individual somatic symptoms between patients with chronic heart failure with depressive comorbidity and those without depressive comorbidity. We hypothesized that depressed patients with CHF would report higher levels of all individual somatic symptoms than nondepressed patients with CHF.

## Methods

### Study design

This cross-sectional analysis of data was conducted with the support of the German Competence Network of Heart Failure (www.knhi.de), a research association funded by the German Ministry of Education and Research (18). Patients were recruited from the cardiac outpatient units of the Department of Cardiology at the University of Heidelberg and Theresien Hospital Mannheim, Germany. The investigation conformed with the principles outlined in the Declaration of Helsinki (19). Ethical approval for the study was obtained from the institutional review board of the University of Heidelberg.

Patients were consecutively recruited from the two centers between July 2004 and October 2006. A study employee (a clinical psychologist) approached patients during their waiting time in the outpatient unit after checking patient charts for inclusion and exclusion criteria (see below). She or he explained the aims of the study to the patients, supported by a short information sheet. The inclusion criteria were as follows: age ≥ 18 years, documented stable CHF, and a New York Heart Association (NYHA) functional class of I-IV. The exclusion criteria were as follows: decompensation of heart failure within the previous 4 weeks, an active listing for heart transplantation, or a psychiatric disorder that limits legal competence. If patients reported being willing to participate, they provided written informed consent.

The dataset assessed in the present study has also been reported in a previously published manuscript about associations between anxiety and self-care behavior in patients with chronic systolic heart failure (20).

### Measures

Comprehensive clinical data, including medication use and medical history concerning cardiovascular risk factors, were assessed according to the Basic Clinical Dataset (BCD) of the Competence Network of Heart Failure (18). Sociodemographic data, including family status, professional training and income level, were obtained from all patients.

Depression severity was assessed using the depression module of the Patient Health Questionnaire (PHQ-9) (21, 22). This questionnaire comprises 9 items, each of which describes a symptom corresponding to one of the 9 DSM-IV diagnostic A criteria for major depressive disorder (21, 23). The main question is “Over the last 2 weeks, how often have you been bothered by any of the following problems?” The response options for each item are “not at all,” “several days,” “more than half the days,” or “nearly every day,” corresponding to scores of 0, 1, 2, and 3, respectively. The summary score ranges from 0 to 27 points, with higher scores indicating greater depression severity. The optimal cutoff point for diagnosing depressive disorder is 10 or greater (24), which was used in our analyses. Thus, for this study, “depressive comorbidity” was defined as a PHQ-9 score ≥ 10. The term “depression” was not used for these patients, as this would require thorough clinical diagnostics, eventually completed by a diagnostic interview.

To evaluate the somatic symptom profile, the Patient Health Questionnaire-15 (PHQ-15) was used. The PHQ-15 was developed as a PHQ module to assess somatic symptom severity and the potential presence of somatization (24, 25). The structure of the PHQ-15 is similar to that of the PHQ-9; it comprises 13 items describing somatic symptoms over the last 2 weeks. The subjects were asked to rate the severity of each symptom as 0 (“not bothered at all”), 1 (“bothered a little”), or 2 (“bothered a lot”). Two additional physical symptoms, namely, feeling tired or having little energy and trouble sleeping, are included in the PHQ-9. Thus, when determining the PHQ-15 score, each individual symptom is coded as 0, 1, or 2, and the total score ranges from 0 to 30 (25). A cutoff point of 10 is recommended for diagnosing moderate somatic symptoms (24). In our analyses, the individual items of the PHQ-15 were also used.

The PHQ-9 and the PHQ-15 were included only in further analyses if fewer than 20% of the items were missing. This approach is consistent with the recommendations of the authors of both questionnaires (24).

A clinical psychologist provided a brief introduction of the questionnaires to the patients. The patients then completed the questionnaires in a quiet area while waiting to undergo their examinations. To prevent time pressure, the patients were permitted to complete the questionnaires at home, and the questionnaires were then sent to the clinic in a stamped envelope. The study physician assigned multimorbidity ratings using the Cumulative Illness Rating Scale (CIRS-G) to estimate each patient’s morbidity burden (range 0-70, with higher scores indicating a greater disease burden) (26, 27).

### Statistical analyses

Sociodemographic and comorbidity data are reported using frequency analyses for categorical data and descriptive analyses for continuous data. To test for differences between patients with and without depressive comorbidity, t tests were conducted for continuous variables with a normal distribution, the Mann−Whitney U test was used for data without a normal distribution, and chi-square tests were conducted for categorical variables.

To assess differences in the distribution of education status and NYHA functional class between patients with and without depressive comorbidity, the Jonckheere–Terpstra trend test was applied to account for small cell sizes.

To compare the severity of individual somatic symptoms between depressed and nondepressed patients, we conducted item-level analyses of covariance (ANCOVAs) based on the 13 items of the PHQ-15. We excluded the two items “feeling tired or having little energy” and “trouble sleeping” because these items are also included in the PHQ-9 depression scale. Age and sex were controlled for, as were the CIRS-G summary score and left ventricular ejection fraction (LVEF), which are objective measures of heart failure severity.

All analyses were conducted using SPSS 27.0.

## Results

### Patient characteristics

Of the 394 patients invited to participate, 47 declined participation because of a lack of time or due to participation in other conflicting studies. Thus, 347 patients were included. Forty patients who failed to complete all the questionnaires were excluded.

Patient characteristics are summarized in **Table 1** for patients with and without depressive comorbidity. The population was predominantly male, with an age range of 19-90 years. The distribution of patients with/without depressive comorbidity into four age groups was as follows [n (%)]: 19-30 years 1 (1.1)/1 (0.5); 31-50 years 16 (17.6)/25 (11.8); 51-70 years 47 (51.6)/115 (54.2); and over 70 years 27 (29.7)/71 (33.5).

**Table 1:** Sociodemographic and clinical data for patients with CHF with and without depressive comorbidity.

	^£^Patients with CHF and depressive comorbidity N=93 (30.3%)	^§^Patients with CHF without depressive comorbidity N=214 (69.7%)	Test statistics*	p value
Age, years (M ± SD)	60.5 ± 14.0	64.2 ± 11.5	1.56	.120
Gender, male n (%)	69 (75.8)	159 (74.6)	0.05	.828
Living with partner, n (%)	59 (67.8)	169 (82.0)	7.17	**.007**
Level of education, n (%)			0.023^§^	.982
No graduation	3 (3.4)	6 (3.0)		
Primary school	71 (80.7)	164 (81.6)		
Secondary school or higher	14 (15.9)	31 (15.4)		
LVEF, % (M ± SD)	31.7 ± 10.8	31.0 ± 11.0	-0.517	.605
NYHA functional class, n (%)			4.125^§^	**<.001**
I	1 (1.1)	12 (5.8)		
II	34 (37.8)	120 (57.7)		
III	53 (58.9)	75 (36.1)		
IV	2 (2.2)	1 (0.5)		
Hypertension, n (%)	64 (70.3)	149 (70.3)	.105	.949
Coronary artery disease, n (%)	56 (61.5)	122 (57.3)	2.984	.225
Atrial fibrillation, n (%)	17 (18.7)	37 (17.5)	.066	.798
ICD, n (%)	31 (34.1)	86 (41.0)	1.27	.260
History of resuscitation, n (%)	7 (7.7)	21 (10.1)	0.45	.504
PHQ-9 depression severity, M ± SD	14.2 ± 3.7	4.5 ± 2.6	-23.12	**<.001**
PHQ-15 overall somatic symptom severity, M ± SD	12.4 ± 5.1	7.6 ± 4.4	-8.40	**<.001**
Multimorbidity CIRS-G, M ± SD (possible range: 0-70^+^)	12.7 ± 8.1	11.2 ± 8.6	-1.47	.142
Antidepressant medication, n (%)	7 (7.8)	4 (2.0)	^#^	**.038**
Contacts with GP within last 12 months (M ± SD)	8.1 ± 9.5	6.7 ± 9.8	-1.92^$^	.055

^£^ Depression severity as measured with the Patient Health Questionnaire (PHQ-9) score ≥ 10.

^§^ Depression severity as measured with the Patient Health Questionnaire (PHQ-9) score < 10.

*T value for comparison of continuous data; X^2^ for comparison of categorical data; Fisher’s exact test was applied given a cell size < 5.

^§^ The Jonckheere-Terpstra trend test for small cell sizes was used to compare differences in the level of education and the distribution of the NYHA functional class between patients with and without depressive comorbidity. The standardized test statistic is given here.

^+^a higher score indicates a higher level of multimorbidity.

^#^not applicable, as Fisher´s exact test has no formal test statistic.

^$^ The Mann-Whitney-U test for data with no normal distribution.

M, Mean; SD, Standard deviation.

LVEF, Left ventricular ejection fraction; NYHA, New York Heart Association; ICD, Implantable cardioverter-defibrillator; CIRS, Cumulative illness rating scale; PHQ-9, Patient Health Questionnaire 9; PHQ-15, Patient Health Questionnaire 15; GP, General practitioner.Bold values means p value <.05.

Approximately one-third of the patients had a positive depression screening. There were no significant differences between depressed and nondepressed patients with regard to age, sex, educational status, LVEF, multimorbidity or number of contacts with the primary care physician.

A greater number of patients without depressive comorbidity were living with a partner than patients with depressive comorbidity. A greater number of patients with depressive comorbidity were classified as having higher NYHA class scores than patients without depressive comorbidity. With an altogether low prescription rate of antidepressant medication, a slightly greater number of patients with depressive comorbidity took antidepressants than nondepressed patients.

Depressed patients had greater total somatic symptom severity scores than nondepressed patients.

### Somatic symptom patterns in patients with and without depressive comorbidity

Patients with CHF and depressive comorbidity scored significantly higher on ten out of the thirteen PHQ-15 items than patients with CHF without depressive comorbidity (**Table 2**). A large effect size was shown for the difference in palpitations between patients with and without depressive comorbidity. Differences in symptoms of headache, chest pain, and shortness of breath had medium effect sizes (0.71-0.77). Small effect sizes (0.31-0.47) were observed for dizziness, nausea, gas or indigestion, and pain symptoms, with the exception of abdominal pain.

**Table 2:** Comparison of patients with CHF with and without depressive comorbidity.

n	PHQ-15 items*	^£^Patients with CHF and depressive comorbidity n=93	^§^Patients with CHF without depressive comorbidity n=214	Test statistic		
		M (SD)	M (SD)	ES	F (5,302)	p
1	**Feeling your heart pound or race**	**1.18 (0.08)**	**0.60 (0.05)**	**0.80**	**39.50**	**<.001**
2	**Shortness of breath**	**1.53 (0.08)**	**0.94 (0.05)**	**0.77**	**39.75**	**<.001**
3	**Headaches**	**0.62 (0.06)**	**0.22 (0.04)**	**0.74**	**29.86**	**<.001**
4	**Chest pain**	**1.03 (0.08)**	**0.51 (0.05)**	**0.71**	**29.27**	**<.001**
5	**Dizziness**	**0.91 (0.08)**	**0.59 (0.05)**	**0.47**	**11.19**	**.001**
6	**Fainting spells**	**0.27 (0.05)**	**0.07 (0.03)**	**0.46**	**11.29**	**.001**
7	**Nausea, gas, or indigestion**	**0.74 (0.08)**	**0.42 (0.05)**	**0.45**	**11.26**	**.001**
8	**Pain in your arms, legs, or joints (knees, hips, etc.)**	**1.13 (0.09)**	**0.86 (0.06)**	**0.32**	**5.77**	**.017**
9	**Pain or problems during sexual intercourse**	**0.58 (0.09)**	**0.34 (0.06)**	**0.31**	**4.43**	**.037**
10	**Back pain**	**0.98 (0.09)**	**0.74 (0.06)**	**0.31**	**5.32**	**.022**
11	Constipation, loose bowels, or diarrhea	0.63 (0.08)	0.49 (0.05)	0.19	1.89	.171
12	Stomach pain	0.32 (0.06)	0.24 (0.04)	0.16	1.29	.257
13	Menstrual cramps or other problems with your periods	0.06 (0.03)	0.04 (0.02)	0.11	0.39	.532

*13 items, as 2 items from the PHQ-9 were excluded.

^£^Depression severity as measured with the Patient Health Questionnaire (PHQ-9) score ≥ 10.

^§^Depression severity as measured with the Patient Health Questionnaire (PHQ-9) score < 10.

n, item, number of the PHQ-15; PHQ-15 Patient Health Questionnaire 15; M, adjusted means; SD, standard deviation; ES, effect size; F, F statistics; p, p value.

Effect size is the difference between the adjusted means divided by the standard deviation of the respective item, in the total sample.

Degrees of freedom in entire patient sample; due to missing data, degrees of freedom vary between df=295 and df=304.Bold values means p value <.05.

## Discussion

According to the present cross-sectional analysis of the data, patients with CHF and depressive comorbidity had substantially greater scores for most but not all of the perceived somatic symptoms than patients with CHF without depressive comorbidity. This finding is partially different from our hypothesis, as we assumed a greater symptom load in all somatic symptom dimensions. The largest differences between patients with and without depressive comorbidity were found for the symptoms “feeling your heart pound or race”, “shortness of breath”, “chest pain”, and “headaches”. The first three symptoms could be attributed to worsening of the underlying cardiac disease. No significant differences between depressed and nondepressed patients were detected in terms of symptoms, such as “stomach pain”, “menstrual cramps”, or “constipation, loose bowels and diarrhea”, i.e., symptoms not attributed to a cardiac disease. On a small but significant level, depressed patients had more symptoms of “dizziness”, “fainting spells”, “nausea, gas or indigestion”, and pain, with the exception of stomach pain, than nondepressed patients.

Our findings confirm those of previous studies that revealed a greater cardiac symptom load in depressed patients than in nondepressed patients with CHF (12). Moreover, we observed much smaller differences or no differences between patients with CHF with and without depressive comorbidity with regard to somatic symptoms that are not or are only scarcely attributed to cardiac disease.

These results cannot be attributed to the greater incidence of somatic disease in depressed patients than in nondepressed patients, as the patients with depressive comorbidity did not differ from patients without depressive comorbidity with regard to the objective severity of heart failure or somatic comorbidities.

Patients with chronic heart failure face a dilemma: guidelines strongly advise patients to perform self-care measurements, such as daily weight control and checking their ankles for fluid retention (1). Thus, symptom perception, which includes “body listening, … as well as interpretation and labeling of symptoms”, is highly recommended (28). This monitoring is meant to be helpful in maintaining a stable condition and preventing cardiac decompensation. Moreover, symptom perception, as an almost obligatory component of the treatment regimen, can be a constant reminder of an underlying and potentially threatening somatic condition. Organic heart disease itself, together with recommended symptom awareness, is a vulnerability factor for bodily distress (15). As patients with depressive disorders frequently present with somatic symptoms in the health care system (8–10), depressive comorbidity in CHF patients could amplify high body awareness and result in a greater cardiac symptom load in depressed patients than in nondepressed patients. Interestingly, and perhaps irritating at first, no clear relationship between depressive disorder and heart failure symptom perception was found in a recent review (28). One possible explanation for these findings could be that depression symptoms, such as rumination and restlessness, could further amplify the perception of bodily symptoms, while depression symptoms, such as difficulty concentrating and feelings of worthlessness, might hamper it. Another effect of body symptom awareness demanded by guidelines may be that depressed patients do not knowingly report more symptoms per se but experience their body symptoms as more burdensome than nondepressed patients. Thus, the presence of heart disease with a depressive comorbidity might lead to a culmination effect with greater impairment by somatic symptoms (15). The clinical consequences could be profound, as patients’ reports of a greater burden of chest pain, breathlessness, and palpitations are more likely to result in medical diagnostic examinations than in the diagnostic evaluation of a potential depressive comorbidity and subsequent supportive actions.

We found depressive comorbidity in approximately 30% of our patients, which is a greater percentage than that reported in the literature (1, 7). This could be due to the younger age of our patient group than in other studies (1, 7). Then, we used a cutoff point of 10 for the PHQ-9, which is recommended for screening purposes (24).

Patients with depressive comorbidity in our study were less likely to be living with a partner than patients without depressive comorbidity. A nonsupportive partnership and a lack of social support are risk factors for the development and course of cardiovascular diseases (7, 29). Thus, the perception and management of somatic symptoms can differ with regard to the presence or absence of social support.

Additionally, patients with depressive comorbidity were in higher NYHA classes than patients without depressive comorbidity, which could be interpreted as an impact of CHF severity on the prevalence of depression. In contrast to objective measures of heart failure severity, such as LEVF or natriuretic peptides, the NYHA class is more strongly associated with other clinical factors, such as depression (30). Accordingly, depressed patients might be rated by physicians as having a higher NYHA class than nondepressed patients due to depression symptoms (little interest, depressed mood, etc.).

If depressive comorbidity is associated with somatic symptoms, why are only some symptom areas depicted in the PHQ-15 elevated in our study? Although patients with depression frequently complain about somatic symptoms, there is no single typical somatic symptom for depressive disorders (8, 9, 11). Rather, depressed patients show symptoms, including sleep disturbances, fatigue and musculoskeletal or back pain; have vague somatic complaints; and have a high number of single somatic symptoms (8, 9, 11). We did not analyze potential differences in fatigue or trouble sleeping between depressed and nondepressed patients, as these symptoms were used in the diagnostic depression algorithm of the PHQ-9. Depressed patients reported a greater burden due to musculoskeletal or back pain than nondepressed patients in our study, even when the difference had only a small effect size. This difference in pain symptoms was unlikely to be due to increased somatic comorbidities because there was no significant difference between depressed and nondepressed patients with regard to multimorbidity, as evaluated by the respective multimorbidity scale. As previously mentioned, patients with depressive disorders tend to report a greater number of somatic symptoms than patients without depression, regardless of the presence of somatic disease (8, 9, 11). Then, depressive comorbidity with a somatic disease can culminate in more somatic symptom complaints (15). As patients with CHF are strongly advised to be aware of symptoms of potentially worsening heart failure according to the respective guidelines (1), they are virtually conditioned for potential cardiac symptoms. Depressive comorbidity might amplify this effect.

### Limitations

Some limitations of this observational study must be stated. The study was cross-sectional, which did not allow conclusions regarding causation to be drawn. Furthermore, the patients were mostly recruited from a tertiary care center, thus limiting the representativeness of the patient sample and thus the generalizability of the data. The patients in our study were younger and more often male than CHF patients in the general population. The etiology of heart failure should be comparable, as approximately two-thirds of our patients had hypertension and/or coronary artery disease. (1). Additionally, the data were self-reported. Self-rating of symptoms can lead to an exaggeration or understatement of symptoms and, with regard to physical symptoms, does not allow for an allocation to a specific cause.

We included patients in NYHA class I-IV but could only gather data from three patients in NYHA class IV. Thus, the transfer from our results to other patients from this severely ill group can only be limited.

We only have limited information about the duration of chronic heart failure, as a clear onset of the disease often cannot be defined. Thus, it was not possible to analyze potential associations between heart failure duration and somatic symptom awareness and depressive symptoms.

Depressive comorbidity was not diagnosed based on a clinical diagnostic interview, so we do not have exact numbers of patients with clinically diagnosed depression.

On the other hand, we included a well-described sample of 307 patients, carefully selected predictor variables, and used established instruments to assess depressive comorbidity and somatic symptoms.

Finally, the data were assessed approximately 15 years ago. Therefore, potential changes with regard to the relevance of depressive comorbidity or treatment regimens during this period could have an impact on the prevalence and treatment of mental comorbidities. However, while recommendations regarding multidisciplinary management have become more prominent in recent guidelines (1, 7), the prevalence of depressive comorbidity has not declined (2, 7), and medical therapy has not fundamentally changed (1). An increase in therapeutic options for mental disorders, e.g., the use of antidepressant medication, is also unlikely due to disappointing evidence, potential side effects, or interactions with cardiac medication (7).

## Conclusions

In a large group of outpatients with chronic heart failure, somatic symptoms were more pronounced in those with depressive comorbidity than in those without depressive comorbidity. This finding especially accounts for cardiac symptoms despite no difference in the severity of the underlying heart disease. The potential interpretation of somatic symptoms as correlates of depressive comorbidity should be considered in routine clinical practice. This approach provides the opportunity to prevent misinterpretation and unnecessary medical diagnostic tests and to offer specific psychosocial support for patients who need it. To ensure the presence of a relevant depressive comorbidity, specific questions, eventually completed by validated screening questionnaires, are recommended in clinical practice in the respective guidelines (1, 7).

## Data availability statement

The raw data supporting the conclusions of this article will be made available by the authors, without undue reservation.

## Ethics statement

The studies involving humans were approved by institutional review board of the University of Heidelberg. The studies were conducted in accordance with the local legislation and institutional requirements. The participants provided their written informed consent to participate in this study.

## Author contributions

TM: Conceptualization, Data curation, Formal Analysis, Funding acquisition, Methodology, Writing – original draft, Writing – review & editing. BL: Conceptualization, Formal Analysis, Methodology, Supervision, Writing – review & editing. LF: Writing – review & editing. NF: Funding acquisition, Supervision, Writing – review & editing. MH: Conceptualization, Resources, Writing – review & editing. HF: Conceptualization, Funding acquisition, Supervision, Writing – review & editing.
